# Turning the Tables

**Published:** 1888-11

**Authors:** 


### TURNING THE TABLES.

In the face of the effort which is now being made in far away Japan, to unite under a single management or endeavor, the missions of the Congregational and Presbyterian establishments, and perhaps other like organized religious systems, whose shades of difference are not easily comprehended, except by over nice sectaries, it is a significant fact that some enterprising Japanese Buddhists have undertaken a missionary work to be prosecuted among Christians, with a view to their conversion to the Buddhistic philosophy and faith, not to say religion. This desideratum is sought to be accomplished by a series of publications in English, explanatory of Buddhism, to be circulated in this country, wherein the footing already supposed to have been gained by the Theoosiphists, is viewed as an encouraging feature.

Some time ago we had our attention drawn to the photograph of a modern brick mansion of ample proportions, and a rather diminutive place of worship, with an meagre brick foundation, standing side by side in the vicinity of Tokio. It was explained that these were two Presbyterian mission homes.

Their history ran as follows: Some years ago, the home agents of the mission, after much importuning, succeeded in collecting funds enough to build a mission church in Japan, and to this end, shipped a quantity of brick and house materials deemed sufficient for the purpose. In due time the mission representative of the Lamb informed his backers that he had caused to be constructed an ample house of worship, and out of the few remaining bricks, a humble dwelling for himself. When the photograph was taken, this humble dwelling had grown to be a stately mansion, beside which, the "ample house of worship," built chiefly of bamboo, looked like a garden toy house. It is to be hoped that under the united effort about to be made, the dwelling will not overshadow the church.

With such an example of Christian endeavor before them, is it at all strange that the adherents of one of the oldest and most humane of prevailing faiths, which has survived the vicissitudes of time and the centuries of opposition of a cunning and powerful priesthood should, from an inborn sense of duty seek to spread abroad among Christian nations, a system of religion wherein self negation and the sacrifice of all personal comforts to the one object of alleviating the sufferings of mankind, is the one prevailing feature?

In a word, are there not some things concerning which even Christians need Christianizing? Buddha with his wooden bowl, seeking alms for the poor, is by no means a latter day conception.

